# Microglia dynamics in aging-related neurobehavioral and neuroinflammatory diseases

**DOI:** 10.1186/s12974-022-02637-1

**Published:** 2022-11-17

**Authors:** Nima Javanmehr, Kiarash Saleki, Parsa Alijanizadeh, Nima Rezaei

**Affiliations:** 1 Student Research Committee, Babol University of Medical Sciences, Babol, Iran; 2 USERN Office, Babol University of Medical Sciences, Babol, Iran; 3 Research Center for Immunodeficiencies, Children’s Medical Center Hospital, Tehran University of Medical Sciences, Dr. Qarib St, Keshavarz Blvd, Tehran, 14194 Iran; 4 Department of Immunology, School of Medicine, Tehran University of Medical Sciences, Tehran, Iran; 5 Network of Immunity in Infection, Malignancy and Autoimmunity (NIIMA), Universal Scientific Education and Research Network (USERN), Tehran, Iran

**Keywords:** Microglia, Neuroimmunology, Neuroinflammation, Neurological disorders, Microglia dynamics

## Abstract

Microglia represent the first line of immune feedback in the brain. Beyond immune surveillance, they are essential for maintaining brain homeostasis. Recent research has revealed the microglial cells' spatiotemporal heterogeneity based on their local and time-based functions in brain trauma or disease when homeostasis is disrupted. Distinct "microglial signatures" have been recorded in physiological states and brain injuries, with discrete or sometimes overlapping pro- and anti-inflammatory functions. Microglia are involved in the neurological repair processes, such as neurovascular unit restoration and synaptic plasticity, and manage the extent of the damage due to their phenotype switching. The versatility of cellular phenotypes beyond the classical M1/M2 classification, as well as the double-edge actions of microglia in neurodegeneration, indicate the need for further exploration of microglial cell dynamics and their contribution to neurodegenerative processes. This review discusses the homeostatic functions of different microglial subsets focusing on neuropathological conditions. Also, we address the feasibility of targeting microglia as a therapeutic strategy in neurodegenerative diseases.

## Introduction

Neurons and glial cells, such as microglia, astrocytes, and oligodendrocytes, are the main cell populations in the brain. Microglia are the dominant immune cells in the central nervous system (CNS), making up to 16% of the human brain's parenchymal cell density [1]. Microglia carry out immune surveillance throughout the neuraxis by responding to pathogen-associated molecular patterns (PAMP) and damage-associated molecular patterns (DAMP) and alleviating the CNS injuries by the phagocytosis of debris and secreting neurotrophic factors [2]. Also, these cells modify neuronal networks by synaptic pruning and maintaining synaptic plasticity throughout life. Thus, microglia contribute to proper CNS integrity in both the healthy brain and neuropathologies [3]. Microglia are the main constituent of the innate immune system in the brain, affecting CNS function and governing neuroinflammation [4]. For instance, infections, trauma [5], ischemia [6], and toxins [7] lead to robust activation of microglia. However, dysregulation of the activity of proinflammatory microglia and other activated cells, including astrocytes and infiltrating immune cells, predisposes the brain tissue to chronic inflammation, which lays a foundation for neurodegeneration [8].

Protracted or excess microglial response via the release of inflammatory mediators potentially exacerbates brain damage. It contributes to progressive neuronal dysfunction [9] and, in particular, neurodegeneration, which is fundamental to the pathophysiology of conditions such as Alzheimer's disease (AD), Parkinson's disease (PD), amyotrophic lateral sclerosis (ALS), and multiple sclerosis (MS) [10, 11]. Nonetheless, microglia can both resolve and exacerbate neuroinflammation. Researchers have focused on the dynamic shift across microglial subpopulations to address the double-edged actions of microglia in the brain [10, 12]. These phenotypical and functional alterations among microglia subsets have been documented by transcriptomic techniques in CNS conditions, such as neurodegeneration [13], neuropsychiatric disease [14], and traumatic brain injury (TBI) [15] across various ages [16] and brain regions (e.g., white matter-associated microglia (WAM) [17]). At the early stages of a neurodegenerative process, an alteration in microglial phenotype and homeostatic markers occurs, leading to the development of disease-associated microglia [16]. These specific microglial subsets could contribute to disease progression through distinct immunopathological pathways [18]. Then, microglia increase their adaptability to the spatiotemporal progression of CNS injuries by constantly changing their profile into pro- and anti-inflammatory states [19]. In sum, gaining insight into dynamics of microglia and their multi-dimensional contribution to homeostatic and disease conditions is critical to obtain optimal diagnosis and therapy in neuropathologies [20].

The technological advances have led to a rapid rise in research on microglia and the possibility of translating the research into the clinic [21]. Here, we discuss microglial heterogeneity in recent scientific advances, with particular emphasis on neurodegenerative disorders. Also, we address the microglia-based diagnostic and therapeutic approaches, particularly those with modulatory effects on microglia behavior in inflammatory conditions.

## Origin, signature, and neurophysiology of microglia

Human microglia originate from mesodermal hematopoietic cells (HSCs), mainly in the yolk sac [22, 23]. By fate-mapping analysis, Ginhoux et al*.* showed that at embryonic week one, primitive myeloid progenitors give rise to microglia, which increase in early childhood [23]. In humans, microglia constitute between 0.5 and 16.6% of glia within the brain parenchyma and are higher in white matter than gray [24].

Transcriptomic analysis revealed a unique microglial profile comprising transmembrane protein 119 (TMEM119), P2Y purinoceptor 12 (P2RY12), Sal-like protein 1 (SALL1), CSF1R, TGFβ1, TGFβ receptor 1 (TGFβR1), and sialic acid-binding immunoglobulin-like lectin H (Siglec-H). Mentioned signatures discriminate microglia from the CNS-associated macrophages (CAMs) and CNS monocytes [25–28]. Microglia produce the classical macrophages' cell exterior biomarkers in the healthy CNS, including F4/80 (EMR1), CD11b, CD45, Iba1, and CX_3_C chemokine receptor 1 (CX_3_CR1), CD200R1, CD172a/SIRPa, and triggering receptor expressed on myeloid cells 2b (TREM2b) [29, 30]. Also, CD45 and CD11b are overexpressed in macrophages compared to microglia, further separating resident microglia from CNS monocytes [26] (Table 1).

**Table 1:** Microglial phenotypes and characteristics

Microglia phenotype		Heterogeneous	Microglia features		References
Activating stimulus	IFN-γ, TNF-α, LPS Pathogen-activated molecular patterns glucocorticoids, danger-associated molecular patterns misfolded proteins, complements	IL-4, IL-13	Immune complexes	IL-10, TGF-β, glucocorticoids	[188–192]
Sources of stimulus	Pathogens, natural killer, T-helper-1 lymphocytes	Apoptotic neurons, granulocytes replying to cell damage, fungi or parasites, T helper 2 cells		Macrophage	[189]
Surface phenotypic markers	iNOS, MHC-II, COX-2, CD14, CD40, CD74, CD86	Arginase-1, Mannose receptor FIZZ-1, chitinase 3-like 3, chemokines and receptor, CD206, CD209, CD200R, CD33, TREM-2	Cyclooxygenase 2, sphingosine kinase, inhibitor of cytokine signaling 3, CD16, CD32, CD64	CD163	[193–195]
Pan markers	HLA-DR, IBA1, CD68	HLA-DR, IBA1, CD68	IBA1, CD68	IBA1, CD68	[194]
Substances produced	Hyperinflammatory cytokines: IL-6/12/17/18/23/1β, TNF-α, ROS, Fibroectin, Prostaglandin E2 Excitatory residue	Extracellular matrix proteins, Fibroectin	IL-1β, IL-6, IL-10, TNF-a	IL-10, TGF-β	[13, 189, 190]
Functions	Hyperinflammatory, present antigen to lymphocytes, phagocytosis, cytotoxicity	Tissue repair, remodeling of extracellular matrix, phagocytosis	T cell recruitment	Anti-inflammatory, phagocytosis	[189],[190]

Possible clinical biomarkers of microglial phenotyping in the CNS. The relevant phenotype biomarkers mentioned should be solely considered as a guide for the identification of microglia but not to restrict the evaluation and taking into account the wide array of activities which various microglial subtypes present within the nervous system. The overview of experiments highlights that a portion of biological markers are utilized in multiple experiments. It is possible that different features of various columns co-exist in microglia

*CSF-1R* colony-stimulating factor-1 receptor, *IBA-1* ionized calcium-binding adaptor molecule-1, *IFN-γ* interferon-gamma, *IL* interleukin, *LPS* lipopolysaccharide, *TGF* transforming growth factor, *TNF* tumor necrosis factor, *TREM-2* triggering receptor expressed by myeloid cells-2, *iNOS* inducible nitric oxide synthase, *MHC* major histocompatibility complex

Microglia play an essential role in CNS development from early childhood to adulthood by inducing apoptosis and synaptic pruning in neurons through reciprocal signaling between CX_3_CL1 (fractalkine). CX_3_CL1 is expressed by CNS neurons and acts as a chemoattractant to immune cells, and CX_3_CR1, its receptor on the microglia surface [31]. Indeed, the knock-down of fractalkine receptor impeded layer-V cortical neuron survival in 7-day-old mice because microglia failed to produce sufficient insulin-like growth factor (IGF). On the contrary, Ueno et al*.* witnessed a disproportionate rise in microglia density proximal to axonal projections of sub-cerebrum and corpus callosum in the postnatal brain in *CX3CR1*-knockout mice. Given the inevitable occurrence of neuropathologies following CX3CR1 blockade, scientists hypothesized that fractalkine communication pathways could be essential for the polarization of microglia into the beneficial trophic subtype, and the absence of this signaling could augment the deleterious inflammatory microglial subtype [32].

In physiological states, the microglia downregulate MHC-I and MHC-II. However, upon stimulation, microglia and CAMs express high levels of MCH-II as well as co-stimulatory antigens, enabling them to interact with APCs and present antigens to T-lymphocytes more efficiently compared to astrocytes, but less efficiently than dendritic cells (DCs) [33]. Furthermore, microglia contribute to inflammation by promoting the synthesis of chemokines, including CCL2, CCL3, CCL4, CCL5, CXCL10, and CCL12, aiding chemoattraction for myeloid and T-lymphocytes [34]. Resting microglia are dynamic with motile filopodia-like processes with different morphologies, while their somata are commonly fixed. Because of their motility, resting microglia constantly carry out immune surveillance throughout the parenchyma and scavenge the brain microenvironment by removing cellular debris, misfolded peptides, and toxic metabolites [35]. Brain injury via DAMPs overproduction perturbs the microenvironment, which contributes to microglial change into robust and dynamic elements of surveillance. Nimmerjahn and colleagues demonstrated that, upon laser injury, resting microglia respond by migrating to the damaged area, as established by the detection of microglial processes around the lesion via two-photon imaging. It implies that the rigid idea of resting microglia might not be complete, as they exert vigilant housekeeper traits [36]. Notably, microglia physiologically communicate with other parenchymal components, such as astrocytes, neurons, and vessels [37]. However, the interaction of microglial processes of adjacent microglia leads to mutual cellular withdrawal [38]. Microglia are the brain's primary immune cells participating in homeostatic feedback [39].

## Microglial phenotypes

### M1/M2 microglia polarization: an outdated concept

Despite the state-of-the-art molecular research undervaluing the classification of microglia as M1 and M2 categories, former literature regarding microglial physiology had widely been centered on this abrogated terminology. Thus, we first debate this concept in brief to draw a more realistic picture of microglial physiology, and we will further discuss its disadvantages to address more novel microglial interactions and metabolic pathways in the following sections [40]. Based on the extracellular circumstances, such as cytokines, lipid mediators, or pattern-recognition receptor (PRR) agonists, microglia can be transformed from the resting state with a characteristically high potency of dynamic surveillance into two subgroups, M1 and M2 microglia, resulting in beneficial or devastating outcomes in the brain [41]. M1 microglia secrete IL-6, IL-12, matrix metalloproteinase-9 (MMP-9), MMP-12, IFN-γ, TNF-α, and other inflammatory components. Thus, they are involved in cytotoxicity, acute inflammatory responses, antigen-presenting cells (APCs) functions, and blood–brain barrier (BBB) permeabilization [42]. M2 microglia express IL-4, IL-10, IL-13, brain-derived neurotrophic factor (BDNF), and TGF-β and participate in wound healing, anti-inflammatory response, phagocytosis of debris, and ECM protection [43]. However, recent transcriptomics data indicate that the previously well-established polarized M1 and M2 classification might fail to fully address microglia's broad array of transcriptional states [44]. Indeed, it is incorrect to describe these highly dynamic cells as "resting/activated" and rigid "M1, M2 pro- and anti-inflammatory microglia". [45]. Microglia in healthy and severed brains express a highly plastic phenotype and function, including tumor-associated microglia (TAMs), WAM, neurodegenerative-phenotype microglia (MGnD), LDAM, and DAM, thus, encouraging researchers to address the temporospatial diversity across the brain in homeostasis and neuropathologies [46].

### Beyond the M1/M2 classification of microglial phenotypes

Microglial implications in brain physiology have gained rising attention over the past decade, and state-of-art research has highlighted the necessity to evaluate broad microglial phenotypes and functions through a wider lens compared to the common concreted M1/M2 perspective. The other subtypes of microglia are classified by expressing a distinct set of surface antigens, applying different metabolic pathways, maturation, and harnessing different functions.

In fact, a growing view suggests microglia have the propensity to transit intracellular metabolic pathways in response to niche triggers, resulting in high adaption potentiality due to microenvironment changes [47]. For instance, scientists, by studying aging mice brain and neurodegenerative disease animal models, has brought attention to other reactive microglia states, including damage-associated microglia (DAM), lipid-accumulating microglia (LDAM), dark microglia, WAM, activated response microglia (ARM), MGnD, and CAM [48].

Critical dampening of glucose catabolism and decreased arterial blood flow in the physiological setting of aging may promote neurodegeneration. Precisely, brain microglial function during aging by consumption of glucose to sustain the inflammatory condition that is an accelerator of neurodegeneration [49]. These age-associated inflammatory microglia express decreased levels of homeostatic genes and higher levels of apolipoprotein E (ApoE) [25]. Transcriptional evaluation of MGnD has demonstrated an increase in ApoE-mediated gene expressions, such as Axl and C-type lectin domain family 7 (*Clec7a*). In contrast, a significant decline has been recorded in another upstream regulator of MGnD, the TGF-β, through downregulation of *Tmem119*, *P2ry12*, myocyte enhancer factor 2A (*Mef2a*), *Olfml3*, and spalt-like transcription factor 1 (*Sall1*). These genes choreograph the lipid catabolism and phagocytic behavior of MGnDs. Moreover, Pimenova and colleagues showed that ApoE-deficient mice and mice expressing TREM2, which facilitate the transition of homeostatic microglia into MGnD, are less vulnerable to AD pathogenesis [50].

Despite the significant variations in the transcription signature of LDAM, DAM, and MGnD, they exhibit similar impairments in phagocytosis and lipid metabolism. This hints at a correlation between these phenotypes' metabolism and function [51]. Dark microglia are named after their dark appearance under the electron microscope. These cells show significant suppression of homeostatic core genes, such as CX3CR1 and P2RY12, and are primarily found in aged brain tissues and AD models [52].

CAMs are classified into three distinct subgroups; first, the meningeal macrophages (MGM), which protect against pathogen invasion. Second, the choroid plexus macrophages that robustly traffic into the brain ventricles. Third, perivascular macrophages (PVM) which protect the BBB integrity and regulate vasoconstriction. Moreover, PVMs serve as a facilitator of the hypothalamic–pituitary–adrenal (HPA) axis by the secretion of prostanoids and prevention of endotheliitis during systemic inflammation [53, 54]. Several phenotypic markers help to distinguish CAMs. In terms of specific surface markers, for instance, all CAMS present CX3CR1, CD11b, and CD45. PVM and MGM overexpress the mannose receptor, CD206. High presentation of the Ly6C, Lymphocyte Antigen 6 Complex, on the CAMs plasma membrane helps to distinguish them from infiltrating monocytes [55]. Compared to microglia, MGM and PVM exhibit major histocompatibility complex (MHC) antigens overexpression and accelerated phagocytosis [56]. PVM protects against infections by increasing the recruitment of white blood cells (WBC) [54]. Experimental evidence from a transient middle cerebral artery occlusion (tMCAO) stroke model demonstrated PVM toxicity through exposure to clodronate (CLO) by intracerebroventricular (ICV) administration of CLO-containing liposomes. The negative impact of PVM depletion is indicated by the disruption of leukocyte chemotaxis, VEGF expression, BBB integrity, and neurological function [57].

Whether DAMs adjacent to injured neurons and myelin aggregates have a protective role or deteriorate neuropathologies is debated [58]. The presence of proinflammatory microglia is confirmed in white and grey matter in different proportions (25% compared to 5%) [59, 60]. Intriguingly, microglia with WAMs' characteristics are exclusively recognized in the aged white matter. WAMs exhibit an increase in the TREM2, crystalline F (CST7)*,* and *Axl.* Single-cell RNA sequencing (scRNA-seq) evidence demonstrated that hypoxia- and phagocytosis-related genes are prominently upregulated in WAMs, enhancing myelin debris breakdown. The WAMs protect the aged brain by breaking down degenerated myelin [61].

Furthermore, compared to DAMs that express AD, PD, and Huntington's disease-associated signature genes, WAMs express high levels of genes mediating atherosclerosis, chemokine signaling, and apoptosis. Interestingly, inducing an immune shift is implicated in vaccinology against both neurological and non-neurological conditions, such as atherosclerosis [62]. TREM2 is expressed on both DAM and WAM. In addition, the expression of ApoE genes is more pronounced in DAM, and WAMs function independently of ApoE. But, in the APP/PS1 early-onset AD mice model, ApoE modulates the function of both WAM and DAM subtypes [59, 63, 64]. Thus, it appears that orchestrating the WAM subtype alters physiological aging compared to AD gene-manipulated aging.

## Determinants of microglial phenotypes and function

Recent research has shown the critical role of microglia in adult brain function. Adult microglia primarily hold the excellent promise of privileged immunity in the CNS [65]. Also, high-throughput RNA sequencing has shown that the microglial phenotype can alter across a broad array of conflicting or overlapping characteristics in the adult brain [21]. The latter prompted researchers to investigate the spatiotemporal and functional sway of the microglia and their modulators.

### Heterogeneity across different brain regions

Spatial heterogeneity of microglia is evaluated by the prevalence and ramifications in multiple brain tissues and by estimating specific surface markers [17]. Through fluorescence tracing and fate-mapping approaches, researchers demonstrated the diversity in morphology and disproportionate presence of microglia in various brain niches; for instance, microglia present in the rostral migratory pathway (RMP) and the olfactory bulb has a high self-renewal capacity [17, 66]. This ability could contribute to post-viral infection olfactory impairments. In this context, a recent experiment revealed that the microglial cells in the olfactory bulb were augmented, while the mature olfactory sensory neurons were decreased in a rodent model of TLR3-regulated upper respiratory tract hyperinflammation. The authors showed that concentrations of the proinflammatory cytokines interleukin (IL)-1β, IL-6, tumor necrosis factor (TNF)-α, and IFN-γ were augmented not only in the nasal mucosa but also in the olfactory bulb of mice administered with the TLR3 inducer poly(I:C) [67]. The ongoing coronavirus disease-2019 (COVID-19) pandemic that is characterized by multi-system involvement (e.g., CNS) highlights the significance of such findings [68–72].

Preclinical studies utilizing scRNA-seq showed that broad functional and phenotypical dynamics could be found in different microglial subgroups in each specific brain region upon exposure to similar factors [73]. Recent research showed an amplified immune reply by microglia of the cerebellum and hippocampus, compared to the response by microglia of the forebrain via analyzing the immune-stimulatory gene expression profiles [74]. The microglia in the striatum and cortical regions show highly efficient chemotaxis to ATP while showing lower phagocytic potency [75]. However, the transcription profile of microglia in the cerebellum exhibits a higher expression of the phagocytic genes associated with the engulfment of apoptotic bodies [76]. Also, the abundance of microglia with impeded phagocytosis and reduced expression of purinoreceptors, which modulate the microglial chemotaxis to the brain regions affected by hyperinflammation, are believed to support the endogenous neurogenesis in the subventricular zone (SVZ) [77].

The extracellular matrix (ECM) components, such as cytokines, vary across brain regions, orchestrating the microglia phenotypes and actions [17, 66]. The evidence depicts a consistent view of the ECM-mediated regional variations in microglia characteristics; however, the precise underlying mechanisms of this heterogeneity are unknown. One hint is that the neuron–microglia interactions manage brain homeostasis and the various communication modalities in different brain areas and conditions [78]. Additionally, the evidence has highlighted the microglia shifting roles of transcription factors, CX3C motif chemokine receptor 1 (CX3CR1), CD200R, sphingosine-1-phosphate receptors, triggering receptor expressed on myeloid cells 2 (TREM2), purinergic receptors, toll-like receptors (TLRs), and non-coding RNAs (e.g., microRNAs) [79, 80].

### Neuronal signaling alters microglial phenotype

Microglial induction is managed through two types of signals from neurons, namely, the "on" and "off" signals [81]. Neuronal "off" signals maintain the resting state in the microglia through the expression of a distinct set of proteins, including CX_3_CL1, CD22, neurotransmitters, and neurotrophins in the healthy neurons. This state is characterized by a low expression of CD68 [82]. The CD200 is a plasma membrane marker on the neuronal surface throughout the neural network, while CD200R, its receptor, is prevalently expressed among microglia cell types. The interaction of CD200–CD200R maintains the resting profile in inactivated microglia. On the other side, neurons in a pathological state constantly show "on" signals, such as upregulation of CCL21 and CCL10 that stimulate microglia proinflammatory activity [83]. In addition to "on" signaling in the CNS, neuronal stress-associated DAMPs (e.g., ATP) and PAMPs (e.g., lipopolysaccharide (LPS)) directly changes resting microglia with potent dynamic surveillance to an active proinflammatory state with upregulated CD68 profile [84]. Loss of "off" signals and the anti-inflammatory microenvironment stimulates the polarization of resting microglia into the neuroprotective microglia subsets, with a potential spillover effect on brain homeostasis [85].

Furthermore, recent studies have shown various functionally different subsets of microglia in neuropathologies, such as DAM [86], WAM [60], MGnD [87], and lipid droplet-accumulating microglia (LDAM) [88], which are discussed in the following sections.

## Immunometabolism governs microglial cellular behavior

Brain homeostasis is highly influenced by the spectrum of functional modes of microglia. Metabolic reprogramming affects microglia's dynamic behavior. In this regard, scientists have suggested function-mediated classifications for microglia instead of the well-established stimulation-mediated profiling as with M1/M2, which could clarify their perceived therapeutic applicability. Research based on M1/M2 classification has led to puzzling and often conflicting findings on how microglia orchestrate CNS physiology, raising important questions.

As discussed, microglia stimulation results in the excessive production of IL-1β, IL-12, prostaglandin, and reactive oxygen species (ROS) upon exposure to inflammatory substances, such as IFN-γ, LPS, and TNF-α. These microglia dynamics show exceptional membrane motility due to phagocytic activity. On the other hand, IL-4 and IL-13 induce a certain dynamic of microglial, resulting in the biosynthesis of anti-inflammatory cytokines and growth factors and boosting stem cell differentiation. ROS and nitric oxide (NO) levels are low in neuroprotective cells and are thus associated with reduced inflammation. Despite the mentioned characteristics, microglia present without such strict behavior in vivo. For instance, a recent study on aged mice highlighted that the IL-4 receptor (IL-4R) declines with age, which is affirmed by a decline in arginase, a characteristic M2 marker. However, they also witnessed a decrease in IL-1β, a specific M1 marker in rodents' brains. Fenn and colleagues recorded a substantial decline in IL-1B in IL-4R knockout mice [89]. In the same line, Zhang et al*.* deciphered arginase-expressing microglia with excess ROS content, a critical proinflammatory component of activated M1 microglia for foreign body degradation, in the theme of aged mice with spinal cord injury [90]. The literature suggests the discordance of the prevalent M1/M2 profiling and underlines a lack of correlation between marker expression and function in pathological brain conditions.

To shed light on these contradictory findings, we summarize the association of microglia, profile alteration, and cellular metabolic pathways. The sway of the Krebs cycle and aerobic glycolysis influences microglial cellular behavior. In fact, the energy-consuming profile of microglia in the setting of hyperinflammation differs from the physiological state. Proinflammatory microglia require imminent energy release and, thus, rely on glycolysis to obtain accelerated rates of ATP production and form vital metabolites such as fatty acid generation and NADPH through hexose monophosphate (HMP) shunt to generate ROS. On the contrary, reparative microglia have gotten play regarding tissue factor transcriptome expression and growth factor synthesis. To sufficiently address these needs, microglia may utilize the tricarboxylic acid (TCA) cycle as a sustained energy source to trigger ATP-mediated tissue repair genes and ROS attenuation. They oxidize cell metabolites to concomitantly generate growth factors, such as polyamines and prolines, and attenuate ROS levels.

Microglia represent a prominent inflammatory profile with accelerated levels of CD11b, iNOS, TNF-α, and IL-6 in response to high-dose glucose substrates (75 in vitro [91]. Interestingly, Fodelianaki and colleagues designated an in vitro experiment by 2-deoxyglucose (2-DG), which is glucose with its hydroxyl residues replaced with hydrogen and thus competitively hampers glycolysis, and witnessed a significant decline in M1 microglia activation upon inhibition of aerobic glycolysis [92]. More to the point, it has been intended that administration of 2-DG, along with LPS, hinders the activating role of LPS in the upregulation of inflammatory cytokines in an LPS-induced PD model. On the same line, glycolysis inhibition by 3-bromopyruvic acid (3-BPA), glucose transporter type 1 (Glut-1) siRNA, and hexokinase (HK) 2 siRNA has exhibited similar neuroprotective results by prohibiting LPS-mediated phosphorylation of mTOR, thus nuclear factor-kappa B (NF-κB) blockade. Recent studies assessed the prominent role of mitochondria in microglia behavior. Mdivi-1 is believed to enhance mitochondrial function and prevent its fragmentation, and its administration dampens microglial proinflammatory cytokine release while preserving microglia subtype markers associated with neuroprotection, such as arg-1 and growth factors [93, 94]. Moreover, following mitochondrial blockade through uncoupling protein 2 (UCP2) regulation, an upsurge of inflammatory cytokine release and gene modulation is observed in primary microglia [95]. One consensus is that even if not at the heart of microglia behavior, mitochondria is partly involved in some activities of microglia, by deriving the microglia toward special dynamics that may be more protective differentiation path. Microglial states and markers are briefly mentioned in Table 2.

**Table 2:** Overview of microglial subtypes markers and features

Type of immune cell	Markers	Promoting factors	Inhibitory factors	Primary function	Condition explored
WAM	Apoe, CD63, Clec7a, TMEM119, CX3CR1, P2RY12,Csf1r [195]	TREM2-dependent-ApoE-independent [196, 197]	CSF1R suppressor [198]	Generates proinflammatory cytokines and display reduced phagocytic activity [196, 199], WAM are precursors to DAM and function in the removal of myelin debris [21, 196]	Aged brain, AD [196]
DAM	Lpl, ApoE, TMEM119, P2RY12, CX3CR1, TREM2, CST7, and Axl [200, 201]	Protein accumulation, apoptotic cells, and myelin debris, and needs signaling via type I interferons (IFN-α and IFN-β (IFN-α/β)) and type II interferon (IFN-γ) [200]	TGF-β and NRROS [202]	DAMs reduce the transcriptional signature exclusive to microglia and enhance IFN-feedback genes, lysosomal genes, genes that code lipid-metabolism elements and further exterior receptors and elements related to the damage of synapses and nerve cells [200]	Aged brain, AD [203], PD [204], ALS [205]
LDAM	RAP1B, RAK1, CHBB, TLR2/4, IFN-γ receptor [88]	TREM2-ApoE pathway, phagosome development, innate inflammation [88], generation of nitric oxide and ROS [206]	Triacsin C [88]	LDAMs are deficient in phagocytosis, generate proinflammatory cytokines [88] and synthesize prominent levels of ROS [206]	Aged brain, AD [51], ALS [207]
MGnD	Clec7a, Lgals3, Gpnmb, Itgax, app1, Fabp5, Ccl2 [208]	Apoptotic nerve cells, relies on the TREM2-APOE pathway [208]	Relies on the TREM2-APOE signaling [208]	This type of microglia loses sensome actions such as TGFβ signaling[209], MGnD microglia might also be protective and constitute an initial reply to nerve cells damage [210]	Aged brain, neuritic dystrophy, AD, MS, ALS [208]

*TMEM119* transmembrane protein 119, *P2RY12* P2Y purinoceptor 12, *SALL1* Sal-like protein 1, *CX3CR1* CX3C chemokine receptor 1, *IBA1* ionized calcium-binding adaptor molecule 1, *TREM2* triggering receptor expressed on myeloid cells 2, *LYVE1* lymphatic vessel endothelial hyaluronan receptor 1, *CCR2* C–C chemokine receptor 2, *P2RY12* purinergic P2 receptors Y12, *Tmem119* transmembrane protein 119, *Siglech* sialic acid-binding Ig-like lectin H, *Gpr34* probable G protein-coupled receptor 34, *MGnD* microglial neurodegenerative phenotype, *LDAM* lipid-droplet-accumulating microglia, *WAM* white matter-associated microglia, *DAM* disease-associated microglia

## Microglia mediate neuroplasticity in aging

Neuroplasticity is the adaptive ability of the brain to alter itself in response to internal or external triggers. Neuroplasticity processes have been categorized into neuroregenerative and function-restoring processes. Microglia engulf cell debris and modify synaptic plasticity throughout life [96]. Utilization of the *Cre-Lox* procedure to suppress microglia resulted in a marked decline in learning-associated synaptogenesis. Furthermore, BDNF released from parenchymal microglia boosts the expression of tropomyosin-related kinase receptor B in neurons, which regulates the synaptic plasticity, demonstrating the prominent microglial role in memory- and learning-associated synapse formation [97].

Neurogenesis, which refers to the formation of new nerve cells in various brain areas, such as the dentate gyrus of the hippocampus and SVZ, is considered neuroplasticity, as it helps the brain's adaptive feedback to new stress by introducing more nerve cells into relevant areas [98]. The link between neural progenitor cell (NPC) induction and hippocampal adult neurogenesis has been established, which is affected by a broad range of physiological factors, including aging, exercise, and psychosocial factors. These conditions are believed to channel their effect through the neuroimmune microglial pathways [99].

A study evaluated the role of ischemic stroke on the activation of neural stem cells (NSCs) in the SVZ. Higher proliferation rates were reported among NSCs extracted from severed brains 14 days following MCAO, as opposed to NSCs isolated from intact brains. They proposed that microglia in NSC cultures played a fundamental role in forming more prominent neurospheres in lesioned brain cultures. Intriguingly, by administering the conditioned medium from injured brain microglia cultures to the naïve brain cultures, the researchers witnessed an acceleration in neurosphere formation, implying the presence of modulatory factors within the medium. Moreover, the NSCs from the severed brain exhibited accelerated differentiation into neurons instead of astrocytes. Future experimentations could determine the effectors modifying the NSCs proliferation following microglia activation in the damaged brain [100]. Preclinical research to decipher the phenotype-specific effect of microglia in NSC activity has also been mainly correlative, comparing microglia density under experimental conditions. However, such studies provide no evidence of microglia's direct and specific regulatory effect on adult neurogenesis.

An ex vivo model was developed to examine microglia's mediation of hippocampal neurogenesis via exploiting transgenic *Csf1r-GFP* mice. This study witnessed increased NPC proliferation among the voluntary running mice compared to the sedentary group. The observed effect was attributed to the beneficial microglia activation, given that the induction of selective apoptosis among microglia in the hippocampal culture hindered the neurogenesis. To obtain a better perspective on the potential links between the microglia and NPC activation, the authors disclosed that sedentary mice hippocampal culture yielded robust NPC activation from latency and neurosphere generation upon transfer of runner mice microglia. To put that into perspective, the data thus far collected confirm that microglia directly orchestrate adult hippocampal neurogenesis induced by voluntary exercise [101].

The effect of microglia on the aging brain has been well-studied. Correlative studies revealed that with aging, a reduction in NPC activation along with a significant rise in microglia load could be found in the hippocampus [102]. By highlighting the proinflammatory attributes of microglia in the aging brain, research explained enhanced NSC proliferation and maturation upon depletion of hippocampal microglia in cultures from 20-month-old mice. The removal of MHC-Il-expressing microglia, which heralds inflammation, contributes to a modest yet significant rise in neurosphere generation frequency in aged runner mice, implying their impeding effect on NSC activity [103]. Given the abundance of MHC II-expressing microglia in the aging brain of mice, the authors intend that the regulatory effects of microglia on the neurosphere formation could be abolished by removing MHC II-expressing microglia [101].

Additionally, fractalkine (CX_3_CL1), a transmembrane chemokine produced by nerve cells such as dentate gyrus neuronal networks, modulates microglia activity via CX_3_CR1, its receptor on the microglia surface, particularly in inflammatory conditions. Studies have presented puzzling and controversial data on how the beneficial or detrimental CX_3_CL1–CX3CR1 signaling pathway influences microglia activity in the brain [104]. For instance, Liu and colleagues utilized CX3CR1 siRNA in a bilateral common carotid artery stenosis (BCAS) rodents model and showed that the CX_3_CL1–CX3CR1 axis worsens the outburst of inflammation in ischemic stroke. The CX3CR1 siRNA significantly hampered microglia activation, white matter lesions, cognitive deficits, and suppressed TNF-α, IL-1β, and IL-6. The authors concluded that the CX_3_CL1/CX3CR1 pathway stimulates the proinflammatory microglial activation during ischemia by p38 mitogen-activated protein kinase (p38MAPK)/protein kinase C (PKC) signaling and inflammatory cytokines [105]. Zhang et al. showed that CX3CR1-knockout mice exhibited dysfunctional microglia activity, leading to exuberant inflammatory cytokines released in neuropathologies [106]. It is well-established that CX_3_CL1 expression declines along with the physiological decrease in NPC activity in the aging brain. Also, both young and aged CX3CR1-deficient mice show reduced basal neurogenesis. Exercise may reverse the physiological age-associated decrease in soluble CX_3_CL1, as brain analysis of mice exercising on running wheels revealed an increase in soluble CX_3_CL1 within the hippocampus of both young and senescent rodents. Further, by administering the CX_3_CL1-blocking antibody into the hippocampal tissue, the scientists witnessed a limited neurosphere formation, impeding the normal post-running beneficial microglial effect on NSC activity. Thus, implying the direct role of the CX_3_CL1–CX3CR1 axis on the mediation of microglia activity [107].

Overall, these results revolutionize the previous notions on the impact of microglia on hippocampal NPC activity and indicate that the CX_3_CL1–CX3CR1 pathway could be essential for dynamic neuron–microglia communication. Ultimately, interventions to modify the CX_3_CL1–CX3CR1 signaling may hold promise for clinically feasible therapeutic approaches focusing on neurogenesis-inducing microglia phenotypes.

## The role of microglia in the neuroimmune mechanisms of CNS disease

Transcriptomic studies indicate that there are three developmental classes for microglia; early, pre-adult, and adult stages. Early microglia upregulate genes associated with cell cycles and differentiation, such as Mcm5 and Dab2. Pre-adult microglia increase the transcription of *Csf1* and *Cxcr2* genes, suggesting neuronal maturation. In adult microglia, the core upregulated genes are Cd14 and Pmepa1, modulating immune reactions [84]. Interestingly, aging extends the discovered subtypes of microglia to a broader spectrum, confirming the new perspective that microglial dynamics are very multidimensional. The microenvironment impacts the gene expression profile of aging brain microglia. However, the age-mediated impact on spatiotemporal dynamics of human microglia at the gene expression stages requires further study [108].

### Neuroinflammation-induced hypoxia

There is a widely debated notion that hypoxia during neuroinflammatory processes is a major contributor to further synaptic distortion, a late complication with a significant impact on learning and cognition. Recently, scientists examined the triggering effect of hypoxia in parallel with LPS on microglia in inflammatory conditions [109]. Utilizing artificial cerebrospinal fluid (aCSF) with O_2_ levels below 10%, they found that hypoxia-induced transient depression of field excitatory postsynaptic potentials (fEPSPs), which usually returns to normal in a short while, exert far more protracted depression of signals with a decline to 66% of the baseline when co-applied with 10 μg/ml LPS to the rat hippocampus. However, LPS alone did not affect basal synaptic transmission and fEPSPs. Through a series of applying alternative concentrations and timings, they confirmed that the long-term depression (LTD) of fEPSPs only occurs by co-administration of hypoxic CSF and LPS. LPS is recognized by the complement receptor 3 (CR3) on the microglial surface and causes accelerated expression of NADPH oxidase on microglia, which releases ROS, in particular, superoxide, into the extracellular milieu [110–112]. Hypoxia affects multiple metabolic pathways within the microglia and accelerates ROS synthesis by NADPH oxidase. In brief, excessive ROS burden leads to the modification of synaptic transmission in surrounding neurons due to the activation of phosphatase 2A (PP2A) within the neurons, and thus endocytosis of AMPAR (A-amino-3-hydroxy-5-methyl-4-isoxazole propionic acid receptor) from post-synaptic surfaces, leading to LTD and neuronal dysfunction in neurodegenerative disorders. By demonstrating the incapability of concomitant LPS and hypoxia in triggering LTD in the CR3 knockout rats, researchers further added weight to the role of microglial CR3 in modifying synaptic properties [109, 113]. Further experimentation on the effect of LTD will refine the current perspective of dementia, particularly regarding memory impairment and synaptic perturbations.

### The role of microglial inflammasome signaling

Neuroinflammation is orchestrated by overexpression of cytokines, mainly the IL-1β, IL-18, and TNF-α. Inducing inflammation by the upregulation of IL-1β is mediated by induction of the cytosolic inflammasome and gene expression of pro-IL-1β through the TLR pathway [114]. The activation of the inflammasome in the myeloid cells, particularly microglia, leads to profound caspase-1 stimulation, accelerating the IL-1β maturation [115–118].

The inflammasome complex comprises multiple interdependent sets of proteins, including NOD-like receptor (NLR) family, pyrin domain-containing 3 (NLRP3) or CARD domain-containing 4 (NLRC4), apoptosis-associated speck-like protein containing a CARD (ASC), adaptor protein, and procaspase-1. These proteins participate in a cascade of reactions, affecting inflammatory cytokine profile [119]. A wide range of microglial stimuli could efficiently activate inflammasomes by attaching to sensor proteins [114]. Interestingly, activated inflammasomes in the microglia are perceived as a primary culprit in the exponential burst of neuroinflammation and consequent neuronal apoptosis or neurodegeneration [120]. Dysfunctional and misfolded protein deposits (e.g., amyloid-β (Aβ) and α-synuclein in AD and PD, respectively) induce NLRP3 protein complex and thus activate the microglial inflammasome [121, 122].

Microglial NLRP3 detects cytosolic stress-related elements, which rise from tissue damage and misfolded protein deposition with the consequential progression of neurological disorders, such as AD, PD, MS, stroke, and TBI [123, 124]. In addition to cellular stress, clinical stress also deserves further study to be linked to microglial cellular alterations, as it also affects inflammasomes and plays a role in neuropsychiatric conditions at the clinical level, even during the perinatal period [125, 126].

Many factors upregulate microglial NLRP3 (e.g., exposure to LPS). Compared to macrophages, the NLRP3-inflammasome activating system is more active in microglia; this is due to the absence of the negative feedback loop of pro-IL-1β upregulation in the microglia [122]. The sustained activity of the NLRP3-inflammasome signaling pathway in the microglia holds potential for developing adverse effects such as chronic inflammation in the brain, which dampens microglial capacity to degrade misfolded protein deposits such as Aβ aggregates, laying a foundation for the AD pathogenesis [127]. NLRP3 signaling pathway persistently activates microglial inflammasomes and potentially controls the microglial functional dynamics, thus leading to neurodegeneration and neuropsychiatric disorders. A better comprehension of inflammasome signaling in microglia could assist the development of therapeutic strategies that target neuroinflammation [128].

## Microglia impairment heralds brain connectivity dysfunction

### Neurobehavioral disorders

Impairment in behavioral items, such as socialization, language skills, intelligence, and limited or repetitive behavioral patterns along with movement disabilities are common traits of neurological disorders, such as autism spectrum disorders (ASD), epilepsy, subtypes of major depressive disorders (MDDs), and schizophrenia [129]. Several hypotheses have attempted to shed light on the pathophysiology of these conditions. Especially for ASD, the central cohesion theory (CC) has been proposed, which attributes the key impairments to a specific perceptual-cognitive theme described as dampened context comprehension. In other words, this theory refers to the inability to "see the bigger picture" in ASDs [130]. Another prevailing theory is the hypo-connectivity model. It intends that remote brain areas (e.g., the parietal and frontal lobes) show underconnectivity, while the local neural networks (e.g., in the frontal lobe) show overconnectivity. The third theory suggests that excitation–inhibition imbalances in the neural network could play a role in ASD [131].

Researchers have recently focused on the implication of neurodevelopmental processes in the course of mental illnesses [129, 132]. Factors predisposing individuals to cognitive disorders include defective synaptic maturation, as shown by impaired functional connectivity in the neocortex. The precise mechanism is not clear-cut, but viewing the cognitive deficits through the lens of genetic disorders has enabled the researchers to identify correlations between the presence of misfolded scaffolding proteins and autism. Also, the autopsy confirmed decelerated transcription of synaptic transmission-related genes among patients with neuropsychiatric disorders [132].

Postmortem examinations corroborated the presence of dysmorphic microglia in the brain of patients with mental diseases. Further, an explanation for these morphological alterations in microglia is primary neuronal impairment. Other scientists believe the cause rests on the influence of environmental antigens on CNS immune replies [133]. Another igniting vision is considering the microglia as the primary culprits in neurobehavioral disorders. As discussed, microglia are the cornerstone in the maturation process of synaptic functional connectivity boosting synaptic quantity, which is markedly attenuated in the pathophysiology of ASD and other cognitive deficit disorders [134].

Many novel studies have focused on the roles of microglial phenotype dynamics in neurodevelopmental conditions. However, the causal relationship remains to be deciphered, whether the microglia activation precedes the neuronal deficits or occurs secondary to neuronal loss [135]. Zhan et al. explored the influence of primary microglia damage on the development of autistic characteristics. A CX_3_CR1-KO mice model was utilized in a recent study, which induces a transient deficit in microglia function, and thereby dampens synaptic elimination. Insufficient synaptic pruning during the early postnatal developmental period results in a significant load of inefficient excitatory synapses. During the first weeks of life in wild-type (WT) rodents, spontaneous excitatory postsynaptic currents (sEPSCs) exert higher amplitudes, mirroring the synaptic sprouting and boosted synaptic multiplicity [136]. In CX_3_CR1-KO rodents, researchers discovered diminished amplitudes of sEPSCs by in vivo LFP coherence and resting-state fMRI signal synchronization compared to WT mice during the same period, implying impaired synaptic multiplicity. They witnessed that insufficient synaptic elimination entails a substantial decline in functional connectivity and undermines synaptic transmission culminating in hindered sociability and amplified repetitive behaviors [137]. These manifestations are interconnected with neurodevelopmental illnesses, such as ASDs and MDDs. Despite the clinical manifestations of hippocampal-mediated mental diseases, controversy remains about the persistency of such defects in the synaptic multiplicity during the following stages of development in CX_3_CR1-KO animals [138].

Together, impaired synaptic pruning affects synaptic numbers and precludes functional brain communications. These results support the idea of the primary role of microglia deficits in circuit-level impairments, neurobehavior, and protracted alterations in brain wiring in neuropsychiatric conditions [138]. Last, the known genetics and environmental triggers of ASDs, such as fragile X syndrome, viral infections, medications, and air pollutants, are involved in the neuropathology of this disease by surfacing existing synaptic pruning impairments [132, 139]. Since maintaining good functional CNS connectivity is pivotal for socialization, it should prompt an evaluation of the prominent role of disparities in synaptic elimination in the presence of a wide range of psychosocial differences and gross brain wirings [140].

### Neurodegenerative diseases

There are many neurodegenerative disorders associated with microglia. Here, we exemplify two of the most prominent examples, MS and AD.

In MS, the CD200–CD200R reactions impact the activation of the microglia. Hoek et al*.* demonstrated that *CD200* null rodents form microglial aggregates rich in CD11b and CD45. The reason for microglia aggregation is attributed to an underlying neuroinflammatory and degenerative cause. They designed an experimental model of MS by myelin oligodendrocyte glycoprotein (MOG)-induced experimental allergic encephalomyelitis (EAE) in *CD200* null mice and witnessed accelerated pathological progression, as opposed to the delayed disease onset in the wild-type C57BL/6 rodents. They recorded enhanced CD68 expression in the brain of CD200 null rodents, implying an excessive microglia population [141].

FcγR expressed on microglia mediates the multitude of their inflammatory activities by attachment of IgG immunoglobulins. This can occur both in the form of endogenous processes and therapeutics. Additionally, FcγR expression rises with normal aging, and in the neurodegenerative processes leads to proinflammatory microglia activation, damaging the neurovascular unit in neurodegenerative diseases. FcγR stimulation activates the signal regulatory protein α (SIRPα), which is produced by a range of CNS resident cells. The SIRPα and its associated ligand on myelin, CD47, in turn, impedes phagocyte engulfment potencies and myelin debris clearance. Intriguingly, Gitik et al. experimented with the CD47-KO mice model of spinal cord injury (SCI) and observed earlier and more extended microglia scavenging response toward myelin debris, leading to enhanced neuronal repair compared to CD47^+/+^ mice. Similar to SCI, MS and other pathologies that involve debris aggregation might benefit from the blockade of SIRPα-mediated phagocytosis blockade. Han and colleagues recorded a reduction in CD47 mRNA in MS plaques, despite its presence in surrounding intact myelin and foamy macrophages. Also, in a CD47 null mice model of autoimmune encephalomyelitis, they found the pathology progression was affected, primarily due to impaired microglia activation upon induction with myelin antigens [142]. Differences in brain area studied, and pathology progressiveness may implicate in the dual function of CD47 in MS.

In AD, cognitive function and behavioral abilities deteriorate. Dementia afflicts nearly 60 million individuals globally, with an incidence rate of 10 million new cases yearly. Approximately 70% of dementia cases are categorized as AD. Fibrillary Aβ aggregation (senile plaques), deposition of neurofibrillary tangles (tau), impaired synaptic plasticity, and degenerative changes are AD characteristics. Moreover, pathological processing of amyloid precursor protein (APP) contributes to senile plaques (SPs) formation, with a consequential rise in free-radicals concentration and inflammation providing a hostile environment for neurons.

Neuroinflammation has become the cornerstone of AD pathology over the past decades. In fact, chronic therapy with non-steroidal anti-inflammatory drugs (NSAIDs) diminishes the risk of AD, demonstrating the prohibitory role of anti-inflammatory medications. Concomitant microglia and astrocytes shedding proinflammatory cytokines accompanied by complement activation orchestrate brain inflammation during AD. Investigations have shown the presence of microglia and mononuclear phagocytes adjacent to SPs in autopsy and rodents with AD. Intriguingly, it has been established that even with Aβ deposition, neurons are spared from inflammatory damage until microglia activation. Microglia actively engulf Aβ fibrils and reinforce their degradation by secretion of inflammatory cytokines, thus averting the disease progression. However, it acts as a double-edged sword because the increased load of cytotoxic factors escalates the risk of local inflammation and AD aggravation. Genetic evidence highlights that the burst of the amyloidogenic Aβ may be the cornerstone for familial AD (e.g., early-onset AD) pathogenesis. Concomitant dysfunctions in APP synthesis and its modification by γ-secretase, presenilin subunits PS1/2 lays a foundation for higher propensity to accumulation in Aβ oligomers [143]. Baik and colleagues demonstrated that in an LPS-induced mouse model of cognitive decline, LPS robustly enhances mTOR and HIF1α transcription [144]. Activated mammalian targets of rapamycin complex 1 (mTORC1) strongly hamper autophagy through phosphorylation of p70S6K [145].

On the other hand, impeded autophagy process and overexpression of Aβ may work in concert to amplify the susceptibility to late-onset neuronal damage and AD manifestations. The mTOR-HIF1α signaling modulates inflammatory cytokines transcription, such as IL-1β and TNF-α. This impact is affirmed by demonstrating the downregulation of IL-1β and TNF-α synthesis upon blockade of the mTOR pathway with rapamycin or metformin [146, 147]. Controversially, Han et al*.* witnessed that activated microglia in mice treated with LPS increased autophagy genes expression and blunt iNOS and IL-6 synthesis, thus increasing cell survival [148]. Aβ administration alters the metabolism of primary microglia. A rise in extracellular acidification rate (ECAR), lactate, and a decline in mitochondrial oxygen consumption rate (OCR) indicate a shift from oxidative phosphorylation to glycolysis [149].

Galectin-3 (Gal-3), an evolutionary conserved multifunctional protein, plays a progressively prominent role in neuroinflammatory processes in AD by orchestrating microglia [150]. Gal-3 is a physiological ligand for TREM2, TLR4, and insulin receptor (IR), influencing a variety of microglial immune response mechanisms, such as BMP and WNT pathways in various neuropathological conditions [151]. TLR4 is a well-known molecule of the innate immunity with versatile effects on the immune response [11]. To further clarify, Gal-3 derives its impact on microglia through IFN-γ and the overexpression of proinflammatory factors by the JAK/STAT signaling. Gals-3–IGFR-1 interplay is considered pivotal in IGF-dependent JAK/STAT signaling and microglial growth. In addition to the triggering effect of Gal-3–TLR4 interaction on neuroinflammation, Gal-3 affects the LPS–TLR4 binding by adhesion to the LPS, thus influencing the downstream inflammatory processes. Gal-3 is needed for proper induction of TLR4 after LPS exposure [152]. LPS-triggered microglial release of neuraminidase, leads to sialic acid destruction within the plasma membrane glycoproteins, which paves the way for phagocytosis augmentation because of the Gal-3 attachment to Mer tyrosine kinase (MerTK) [153].

There are many studies leveraging the Gal-3’s pivotal role in AD pathophysiology [153, 154]. Recent observational studies unraveled a substantial increase in the Gal-3’s serum and CSF level in AD cases compared to the control group [155–157]. An experiment conducted by Tao et al. exhibited a concordant rise in Gal-3 and Aβ oligomerization in the frontal lobe of AD patients [158]. Moreover, Gal-3 concentration is congruous with the cognition decline and AD’s stage. In concert with the AD brains, 5xFAD (familial Alzheimer’s disease) mice disclosed a substantially boosted expression of the Gal-3, particularly in microglia entangled with Aβ fibrils. Interestingly, Gal-3-KO 5xFAD mice exert significantly lower levels of Aβ and improved cognition [159]. Higher concentrations of sTREM2 have been found in the plasma as well as cerebrospinal fluid (CSF) of cases with AD [160, 161]. TREM2 and TLR-centered immune responses in microglia adjacent to Aβ plaques are significantly impeded in Gal3-KO 5xFAD mice. Microglial Gal-3 engages in fibrillary Aβ accumulation and decelerates its destruction. The stimulatory effect of the Aβ on microglial Gal-3 transcription orchestrates the inflammatory process. This occurs in concert with the previously observed Gal-3-dependent α-synuclein-stimulated microglia activation [162, 163]. Further investigation into the potency of Gal-3 inhibition as a novel therapeutic approach to protect against Aβ toxicity is warranted.

To reduce the burden of Aβ aggregates, microglia engulf Aβ deposits. Disturbance in the transcription of the cytokines and phagocytic ligands in microglia provides a solid ground for phagocytosis impairment and Aβ accumulation [164]. Microglia are key regulator and effector in the neuropathology of AD, however, a number of riddles regarding the reaction of microglia and AD are unanswered. Neurodegeneration has been linked to microglia-related inflammatory elements including TNF-α, IFN-γ, and IL-1β. In this condition, microglia cannot endocytose neuropathological Aβ and tau. As a result, Aβ and tau accumulation leads to inflammatory induction, culminating in a vicious cycle in AD neuroimmunopathology. Despite this, anti-inflammatory elements excreted via microglia including IL-2, IL-4, IL-10 and TGF-β, as well as the activation of certain receptors such as TREM2, help the restoration of learning and recollection dysfunctions in AD via various signaling pathways and mechanisms. Additionally, phagocytosis and autophagy of microglia mediated by several key receptors including SR-A and CD36 are responsible for the destruction of accumulated Aβ and tau in AD. Overall, even though there are many plaque-related microglia in the CNS of AD cases as well as in preclinical models of AD, microglia cannot adequately clear Aβ deposits. Interestingly, microglia could be triggered to do so by Aβ immunotherapy/vaccines attributable to anti-Aβ antibody triggering of IgG receptor (FcR)-regulated phagocytic clearing of Aβ plaques [164, 165].

Other than injuring nerve cells' via the phagocytosis of synapses and affecting tau neuropathology, microglia interact with accumulated plaques and expiring nerve cells in a proinflammatory manner, thereby damaging nerve cells via the excretion of inflammation modulators. Aβ plaques/fibrils may function as DAMPs and trigger TLRs and the NRLP3 inflammasome [166], leading to the microglial synthesis of TNF-α, IL-1β, and the rest of the inflammatory cytokines. In line with a pathological function for cytokine secretion, the progression of tau neuropathology in *Cx3cr1*-knockout rodents was inhibited with IL-1 suppression [167], and detrimental influences of apoE4 in the context of tau neuropathology were linked to augmented TNF-α synthesis via microglia in vitro. Gene deletion of NLRP3, caspase-1 and TLRs have been found to improve Aβ accumulation and cognitive impairments in amyloidosis rodent models, corroborating the notion that "classical" neuroinflammation advances AD neuropathogenesis.

Additionally, microglia may collaborate with astrocytes to induce nerve cell damage. Three elements secreted via induced microglia (e.g., IL-1α, TNFα, and C1q) are required and sufficient to trigger astrocytes into a neurotoxic condition named "A1," which leads to nerve cells' expiry [168]. A1 astrocytes are present in tau transgenic rodents producing human apoE4 [169] and in CNS tissue from cases with different neurodegenerative conditions, such as AD. Notably, A1 astrocytes demonstrate robustly activated production of complement proteins C1r, C1s, C3, and C4 [170]. As a result, astrocytes may work together with microglia to regulate complement-reliant neural toxicity.

Inflammasome/complement/autophagy are elements of cellular breakdown mechanisms required to break down further or disfigured particles in lysosomes. Such mechanisms, maintained through a sizable enzymatic breakdown system, are disrupted during senescence and are of particular significance during AD, as demonstrated with the autophagy impairment and the augmentation of autophagosomes in cases with AD [171]. Also, lysosomal acidification and autophagy are insulted via AD-associated PS1 mutation [172]. Research shows that microglial Aβ phagocytosis results in neurodegeneration via activating NLRP3 and lysosomal cathepsin-B, eventually leading to maturation and secretion of IL-1β [173]. Thus, degenerating cellular mechanisms may confer opposing roles through distinct mediation of the inflammasome. It could be defensive in neurophysiological conditions and during the early stage of neuropathology and harmful during long-lasting and late stages of illnesses [174].

## Future insights for therapeutic approaches harnessing microglia subtypes in CNS disease

Therapeutic modulation of the brain myeloid cell subpopulations reflects a robust approach to treating various neurological illnesses. Researchers have recognized multiple candidates, such as high mobility group box 1 (HMGB1), adenosine monophosphate-activated protein kinase (AMPK), peroxisome proliferator-activated receptor gamma (PPARγ), and glycogen synthase kinase 3β (GSK3β). For utilization of these candidates' neurodefensive potential, multiple medications are currently undergoing evaluation.

HMGB1 acts as an inflammatory non-histone chromosome element, and injured nerve cells secrete it, and triggered macrophages release this protein. A major secretion of HMGB1 is found in primary cortex culture following N-methyl-D-aspartate (NMDA)- or glutamate-induced excitotoxicity. Primary microglia cultures exposed to a medium of NMDA-administered primary cortex cells were induced. This was confirmed through NO excretion and inflammatory elements production, TNF-α, cyclooxygenase-2 (COX2), and iNOS. In AD, HMGB1 is linked to senile plaques (SPs) and inhibits microglial Aβ42 clearing, thereby increasing Aβ42 neurotoxicity. Through binding HMGB1/2, the small molecule inflachromene inhibits post-translational alterations and secretion, leading to attenuated microglial induction. Further, glycyrrhizin, one of the triterpenes that is isolated using the roots and rhizomes of the herb *Glycyrrhiza glabra* (licorice), attaches to HMGB1, suppressing chemoattraction and mitogenic actions [175–178].

In preclinical settings, the induction of the AMPK is associated with diminished NF-κB induction and a subsequent reduction in the production of proinflammatory elements or iNOS in glia. A number of organic and artificial agents are well-established AMPK inducers, such as berberine, resveratrol, metformin, and 5-amino-4-imidazole carboxamide riboside. Preclinical research has verified the potential of these elements to produce neural protective influences in a wide array of conditions, such as Aβ-associated neural toxicity [179–181]. A preclinical simulation of MS suggested that the phosphorylated and overall AMPK are attenuated during the disease course while they rise in the recovery epochs. Also, mediation of AMPK signaling occurs after the production of IFN-γ and CCL2 (an IFN-γ-associated cytokine) in the brain [182].

The cannabinoid receptors reflect an excellent aim as they can trigger a change in microglial dynamics that is associated with reparative functions. A lack/absence of CB1/2 production may be recognized in inactive microglia. CB2 receptors are increased in the induced microglia, as has been detected in CNS specimens from cases affected by the neurodegenerative disease [183].

Pharmacological delivery methods, including poly (methyl methacrylate) nanoparticles (PMMA-NPs), are engineered to penetrate especially into induced microglia/macrophages and secrete pharmacologically functional elements, including pioglitazone and minocycline, that have been found to mediate microglia induction in various experimental settings [184]. In fact, a broad spectrum of agents and methods, consisting of rifampicin/rifampicin quinone, candesartan cilexetil, RSLA, TAK-242, JWH133, JNJ7777120, MCC950, AAV2-hIL-10, PDE suppressors (e.g., ibudilast, rolipram), PPAR-γ inducers (e.g., rosiglitazone, pioglitazone), and vitamins (e.g., Vit_D_) provide neurodefensive benefits due to their mediating influences on microglial induction [185, 186].

Ultimately, genetic transportation vehicles aiming at microglia have been evaluated in experimental models to mediate cell induction. To reach this goal, recombinant vectors according to adeno-associated virus (rAAV) as genetic transportation vehicles have been engineered. Genes can be transported into mammals' cells by the rAAV. There, it blends in the short genomic area. To successfully produce microglia-exclusive AAV-originated vectors, scientists should utilize cell-niche-exclusive mammals' promoters, including modulatory factors for human CD11b and CD68 and rodent F4/80 [187].

Immunometabolism effects on microglial activation should also be studied further in the wide range of neurobehavioral and neurodegenerative conditions. The phenotypic categorization of microglia should also be better developed. Finally, microglia should not be studied merely as an isolated subtype of cells. It is helpful to consider the interconnections of α-synuclein–microglial cell, nerve cell–glial cell, astrocyte–microglial cell, and mast cell–glial cell along with the microbiota–gastrointestinal–neural pathway. These interactions are believed to play a role in controlling microglial induction and neural proinflammation.

## Conclusion

Microglia are involved in age-related neurophysiologic and diseased conditions. The neurophysiology of microglia is implicated during CNS development and maturation. Microglia interact with a wide array of neural networks. Moreover, the concept of microglia shift represents a wide range of phenotypes beyond M1/M2 classifications, and its associated harms and protective effects vary across different brain regions and conditions. Primary microglia dysfunctions affect neural connectivity and could lead up to cognitive deterioration. In addition, changes in immunometabolism and hypoxia govern microglia function. These factors deserve further study.

**Fig. 1:**
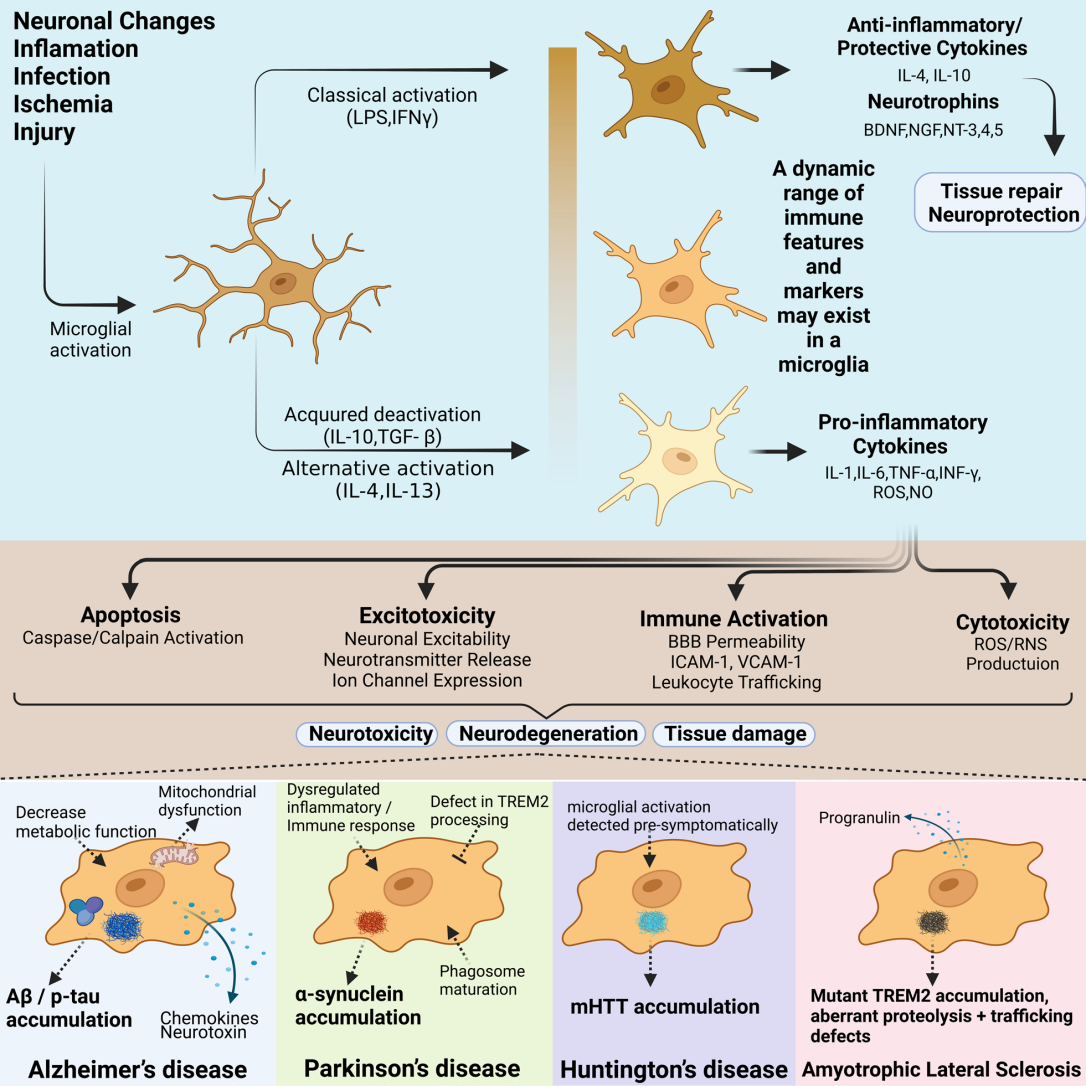
Common mechanisms by which microglial activation and subsequent hyperinflammation may contribute to neurodegeneration and neuropathological mechanisms of AD, PD, HD, and ALS in human microglia. Some microglial cells tend to produce proinflammatory factors, chemokines and neurotoxic elements, whereas the other microglia synthesize anti-inflammatory, neurodefensive and wound-alleviating elements. Microglia may show a mix of these features and should be viewed as dynamic cells in which the features of the old classification co-exist. Proinflammatory cytokines induce cell death, excitotoxicity, immunological induction, as well as cellular toxicity. These effects exacerbate the core neuroimmunological dysregulations in the neurodegenerative disorders. Illustration created with BioRender.com [211]

Moreover, microglia affect neuroplasticity features and adult neurogenesis. Commonly acknowledged proinflammatory or anti-inflammatory characteristics of microglia (Fig. 1) may contribute a quintessential role to neural degeneration and regeneration, respectively. The focus of therapeutic research in preclinical settings is the microglia's adaptation towards a cellular dynamic that leads to improved neurological and neurobehavioral findings both at the molecular and clinical levels. This necessitates a deeper dive into microglia neurophysiology and associated regulatory molecular pathways to open new therapeutic avenues for neurobehavioral disorders.

## Data Availability

Not applicable.
